# Changes in Major Aromatic Compounds of Roasted Coffee Bean Powder During Storage Using Colorimetric Sensor Array

**DOI:** 10.3390/foods15213777

**Published:** 2026-10-22

**Authors:** Jae Kyung Lee, Kyeong Hwan Kim, Robin Park, Jung A Ko, Su Hyun Lee, Hyun Jin Park

**Affiliations:** 1 Department of Biotechnology, College of Life Science and Biotechnology, Korea University, 145 Anam-ro, Seongbuk-gu, Seoul 02841, Republic of Korea; ljk896@naver.com (J.K.L.); kyeohkim@hanmail.net (K.H.K.); elfinja@korea.ac.kr (J.A.K.); 2 Department of Food Bioscience and Technology, College of Life Science and Biotechnology, Korea University, 145 Anam-ro, Seongbuk-gu, Seoul 02841, Republic of Korea; 2022140433@korea.ac.kr; 3 Natural Product Systems Biology Center, KIST Gangneung Institute of Natural Products, Gangneung 25451, Republic of Korea

**Keywords:** Robusta, cross-responsive sensing, chemometrics, headspace volatiles

## Abstract

Aroma is one of the most important factors in the quality of coffee. A colorimetric sensor array (CSA) that gives a pattern of responses to the coffee aroma was used to follow changes during storage. The response of the array to four major aroma compounds of coffee, furfuryl alcohol, dimethyl trisulfide, 2,3-butanedione and 2,3-pentanedione, was examined first, and the four compounds proved distinguishable by their response patterns in hierarchical cluster analysis (HCA). The changes in volatile constituents of coffee during storage at 4 and 25 °C were studied. The response patterns of the stored samples were examined by principal component analysis (PCA). The samples became less distinguishable from one another as the storage period lengthened, converging in the score plots from day 10 at 4 °C and from day 5 at 25 °C. This behavior is consistent with a loss of aroma freshness during storage. GC-MS was used to quantify individual aroma compounds in the stored samples. These results show that the colorimetric sensor array has potential as a rapid indication of the overall state of coffee aroma during storage, complementing the quantitative analysis of individual compounds by GC-MS.

## 1. Introduction

Coffee is consumed worldwide chiefly for its sensory characteristics, above all its aroma and flavor, with health-related considerations a secondary influence on consumption [1]. As consumers’ demands and expectations for coffee have increased and diversified, it has become more necessary to understand the factors that affect the flavor characteristics of coffee. The aroma of coffee, one of the principal determinants of its quality, has accordingly received particular attention [2,3]. Much of this work has addressed the roasting conditions, in particular the roasting temperature and time, because the hundreds of aroma compounds formed during roasting determine the quality of the product. Roasting generates characteristic flavors through pyrolytic reactions [4,5]. Among these components, the sulfur compounds, including sulfides and thiols and in particular 2-furfurylthiol, are one of the groups that contribute most [6,7], as are the Strecker aldehydes methylpropanal, 2-methylbutanal and 3-methylbutanal [3,8,9,10].

Several of these compounds are widely used as indicators of coffee freshness in flavor analysis [2,11]. The Strecker aldehydes named above, and the diketones 2,3-butanedione and 2,3-pentanedione, are among the compounds most frequently followed for this purpose [11,12,13]. Aroma freshness has traditionally been judged by human assessors, and such judgments are subjective. An objective instrumental method is therefore required. Since the earliest changes in aroma freshness are associated with the loss of light volatiles [14,15], the changes in the volatiles during storage were investigated. Such losses are not the only change that takes place during storage. Both the amount and the composition of the aroma may alter with time. How rapidly these changes proceed is expected to depend on the storage temperature, so the same coffee may not develop in the same way under different storage conditions. The aroma of a ground coffee is therefore not a fixed property of the roast, but a state defined by a particular storage time and temperature. Following such changes calls for a method that can monitor the major aroma components rapidly and repeatedly.

Typically, coffee aroma can be analyzed by standard techniques such as liquid chromatography, gas chromatography, and electrochemical techniques [2,16,17,18,19,20]. These techniques separate similar compounds well, but each analysis is time-consuming and requires costly instrumentation and skilled operators, which makes them poorly suited to frequent or on-site monitoring [16,21]. The compounds responsible for coffee aroma are for the most part volatile and unstable. For monitoring of this kind, a rapid method is therefore of more practical use than a slower method of high quantitative precision. Of the approximately 900 volatile components that constitute coffee aroma, about 20 contribute most to its flavor [3,16,22]. Determining the full set of volatile components individually is therefore inefficient. A colorimetric sensor array (CSA), by contrast, detects analytes through the color changes produced by strong interactions between the volatile components of a food and the sensing materials. A CSA is also a simple and efficient means of monitoring freshness, since changes in the principal aroma components can be observed within a short time [17,23]. A CSA is cross-responsive, with each sensing spot responding to a class of compounds rather than to a single molecule [23,24,25]. A CSA formulated for the classes of compounds that dominate coffee aroma can therefore provide an efficient means of following changes in those components.

The discriminating power of a CSA arises from the chemistry of its sensing materials rather than from physical separation. Each spot of the array carries a material that changes color chiefly on contact with a particular class of volatile compounds, and different materials are used for different classes. The array therefore returns a pattern of responses rather than a single signal, so that compounds giving similar responses on one spot can still be separated by the pattern as a whole [24]. Arrays of this kind have been used to follow warmed-over flavor in cooked chicken [23] and to predict key aroma development in coffees roasted to different degrees [17]. Electronic noses have been applied to green coffee beans for comparable purposes [19]. Their response, however, arises mainly from weak physical interactions rather than from the defined chemical reactions used here [17]. Multivariate treatment of coffee aroma data is well established: machine learning has been applied to HS-SPME-GC-MS profiles of oxidized coffee [11], principal component analysis to hyperspectral images of roasted coffee [26], and both principal component analysis and hierarchical cluster analysis to the colorimetric responses described above [17]. Colorimetric sensor arrays have also been used to discriminate the aromas of different coffees [27].

The CSA responses were examined by hierarchical cluster analysis (HCA) to compare the responses obtained from individual aroma compounds, and by principal component analysis (PCA) to describe how the response patterns of samples differing in storage period are arranged. Hierarchical cluster analysis groups objects into clusters on the basis of their similarity. Data sets in a specific cluster share many characteristics, but are very dissimilar to objects not belonging to that cluster. PCA is a method to identify correlated changes in high-dimensional data sets. It is particularly well suited for analyzing the correlated patterns [26,28,29]. Once a data set has been assembled, the position that a measurement occupies within it describes how that measurement is related to the others [25]. These multivariate statistical techniques were used to describe the structure of the data by reducing the number of variables. The contents of the main aroma compounds of the same samples were determined separately by GC-MS.

Whether a CSA can follow the changes that occur in ground coffee during storage has received little attention, and is the question addressed here. The study had two aims. The first was to follow, by CSA, how the aroma of a coffee of fixed roast changes with storage time, and whether that change proceeds differently at two storage temperatures; our earlier study compared coffees roasted to different degrees, each measured at a single point in time, and so treated the aroma as a fixed property of the roast rather than as a state that evolves [17]. The array chemistry is retained unchanged from that study so that the two datasets remain comparable, and the novelty of the present work therefore lies in the application rather than in the chemistry. The second aim was to examine whether such a measurement can address the practical limitations of targeted quantitative analysis and so serve alongside GC-MS, which identifies and quantifies individual compounds.

## 2. Materials and Methods

### 2.1. Sample Preparation and Storage

Robusta green beans, which were harvested in Dak Lak, Vietnam, at an altitude of 600–800 m and processed by the washed method, were purchased from an importer. Coffee roasting was conducted using a Hottop roaster (KN-8828P, Chang Yue Industrial Co., Tainan, Taiwan). Approximately 250 g of green beans were poured into the roasting chamber at 75 °C, as recommended by the manufacturer, and roasted using the automatic roasting program [17]; the beans were ejected when the temperature of the roasting chamber on the display reached 225 °C (dark roast). The roasting was carried out twice under the same program. The roasted beans were cooled under the hood for 30 min at room temperature (25 °C), combined, and finely ground together using a coffee grinder (Super Jolly Electronic, Mazzer Luigi S.R.L., Venice, Italy), so that the material held at the two storage temperatures came from the same pool. The ground coffee was divided into ten portions of 40 g, each sealed in an aluminum package (Hong Jin Co., Seoul, Republic of Korea) fitted with a one-way aroma valve, which releases the carbon dioxide retained by the beans after roasting. Five packages were held in a refrigerator at 4 °C in darkness and five at 25 °C (room temperature) for 25 days. Conditions of the storage environment other than these two temperatures and the light conditions stated lie outside the measurements made. Samples were withdrawn for analysis on days 1, 5, 10, 15, 20 and 25 of storage. Each package was sampled at every one of these points; the material withdrawn was used for the analyses carried out that day and then discarded, and the package was resealed and returned to storage. The five packages held at a given temperature therefore provided five replicate array measurements for each storage time. The array measurement and the GC-MS analysis were both carried out at all six sampling points, the GC-MS determination being made in triplicate.

### 2.2. Determination of Volatile Compounds

#### 2.2.1. Extraction of Volatile Compounds by Headspace Solid-Phase Micro Extraction (HS-SPME)

A 0.1 g portion of ground coffee was placed in a 20 mL headspace vial with a magnetic stir bar. 10 μL of fluorobenzene solution (50 mg/L in methanol) was added as an internal standard, and 6 mL of saturated sodium chloride solution was added. Before the vial was closed with a silicone septum, the inlet was wrapped with polytetrafluoroethylene (PTFE) tape to prevent leakage. Then, an 85 μm Carboxen/polydimethylsiloxane (CAR/PDMS) SPME fiber (Supelco, Bellefonte, PA, USA) was exposed to the headspace of the vial for 30 min at room temperature under stirring. After extraction, the fiber was inserted into the injection port of the GC for 10 min at 230 °C [30].

#### 2.2.2. Quantitative Analysis of Volatile Compounds

Analyses were performed on a model 7890 GC/5977A MS (Agilent Technologies, Santa Clara, CA, USA) with a DB-WAX column (60 m × 320 μm × 0.5 μm, Agilent Technologies) and the carrier gas was helium (flow rate 1 mL/min). Calibration curves were constructed in a blank matrix, which was prepared by driving the volatile constituents off coffee in a vacuum oven at 150 °C for 12 h. A coffee matrix was used rather than solvent so that the standards were presented in a material resembling the samples, in which lipids, melanoidins, polysaccharides and proteins influence how an analyte partitions into the headspace during solid-phase microextraction. The temperature and the duration were chosen so that the volatile constituents were driven off. That temperature lies below the range in which the roast chemistry of coffee is generally described as proceeding: the Maillard reaction during roasting is usually taken to begin at about 160 °C, the formation of acrylamide is greatest between 160 and 180 °C, and roasting itself is carried out at 180 to 240 °C for 5 to 15 min [31]. Twelve hours at 150 °C is a long exposure, and it cannot be excluded that the treatment modified the matrix through further Maillard reaction and thermal degradation rather than only removing volatiles, so the material approximates a blank rather than being one. The same preparation was used for all of the calibration curves, so that the standards for the five compounds were presented in a consistent matrix. Furfuryl alcohol, 2,3-butanedione, 2-methylbutanal, 2,3-pentanedione, and 2-ethylpyrazine were targeted; these five markers were judged sufficient to follow the change in the aroma over storage. Dimethyl trisulfide was not included among them; it served a different purpose, as one of the reference standards used to establish the response of the array to compounds of different chemical classes. A calibration curve was prepared in the range of 1 μg/L to 1000 μg/L for all targeted substances except furfuryl alcohol. In the case of furfuryl alcohol, the calibration curve was covered in the range of 1 mg/L to 100 mg/L. The coefficient of determination of each calibration curve was not less than 0.98. Calibration standards were measured in each analytical batch alongside the samples quantified against them. The retention time of each compound was constant across the analyses, its relative standard deviation not exceeding 1.4%, and the relative standard deviation of the replicate determinations made at each storage time and temperature had a median below 10%.

### 2.3. Fabrication of CSA and Data Collection

#### 2.3.1. Fabrication of CSA

The CSA was prepared in a 3 × 4 array. The array is divided into two groups. The top six are composed of sensors that react with carbonyl compounds such as aldehydes and ketones. The reagent used for this group was 2,4-dinitrophenylhydrazine (DNPH), which forms hydrazones with aldehydes and ketones. Six pH indicators were used for this group: thymol blue, cresol red, bromocresol purple, neutral red, bromoxylenol blue, and metanil yellow. Each of these pH indicators was then mixed with DNPH and polyethylene glycol (PEG)-400 in a ratio of 1:3:3. The other six sensors are those that react with sulfur compounds, such as thiols and sulfides. The reagent used for this group was 5,5′-dithiobis(2-nitrobenzoic acid) (DTNB), which undergoes thiol–disulfide exchange with free thiols. Six further pH indicators were used for this group: bromothymol blue, cresol red, bromoxylenol blue, bromophenol blue, chlorophenol red, and metanil yellow. Each was added to a mixed solution of DTNB, PEG-400 and 0.1 N NaOH, so that the ratios of pH indicator/DTNB/PEG-400/0.1 N NaOH were 1:3:3:1. Both ratios refer to the stock solutions. They were established in preliminary experiments carried out during the development of the array, in which different proportions of pH indicator, reagent and PEG-400 were compared, and have been retained unchanged so that the responses remain comparable across datasets [17,23]. PEG-400 is included to stabilize the sensing materials on the membrane [23]. To fabricate the CSA, the twelve sensing materials were printed on a polyvinylidene fluoride (PVDF) membrane using a micropipette, 5 μL of each being deposited at its position so that the size of a spot is set by that volume and by the membrane. The array was used after drying for 30 min under the hood [17]. A separate array was prepared for every measurement.

The stock solutions were prepared as described previously [17]. For the DNPH stock solution, approximately 1.0 g of DNPH was dissolved in a mixture of 3 mL of water and 10 mL of ethanol. Then 5 mL of concentrated sulfuric acid was added dropwise during magnetic stirring, and the solution was stirred for a further 10 min and filtered through filter paper. Each pH indicator stock solution was prepared by dissolving approximately 5 mg of the indicator in 10 mL of ethanol. For the PEG-400 stock solution, approximately 2.0 g of PEG-400 was dissolved in ethanol, and the volume was adjusted to 20 mL. For the DTNB stock solution, approximately 5.0 mg of DTNB was dissolved in 5 mL of phosphate buffer (pH 7.0).

#### 2.3.2. Data Acquisition

An online monitoring instrument with Color Analysis software was used [17,23]. A 3.0 g portion of sample was transferred to the sample chamber, and after the sample had been set in place, the volatile components were allowed to saturate for 5 min. At the same time, the printed array was placed in the reaction chamber and left for 3 min to stabilize under a nitrogen flow of 300 mL/min. Since the CSA device has a three-way valve structure, both operations could proceed simultaneously. Then, the valve was opened to allow the nitrogen gas to reach the sample chamber and the reaction chamber sequentially. The array in the reaction chamber then responded to the coffee volatiles carried by the nitrogen stream. The color changes were monitored in real time by the camera located at the top of the CSA, the imaging conditions being held constant throughout so that the difference values are comparable between measurements. Color Analysis software was used to extract color values (R, red; G, green; and B, blue) from the captured CSA images. Averaged color values were extracted from the center of each sensing spot in a collected image. Subtracting the initial image from the final exposure image (ΔR, Rf–Ri; ΔG, Gf–Gi; and ΔB, Bf–Bi) provides a color change profile for the coffee aroma. Because each array is referred to its own initial image in this way, differences in the initial color of the sensing spots between arrays, or between preparation batches, do not enter the analyzed response. Ten images were taken at intervals of 30 s, and images were obtained by observing changes for 5 min. The sample portion, the saturation and stabilization times, the nitrogen flow rate, the imaging conditions and the referencing of each array to its own initial image were the same for every measurement reported. Each measurement therefore produced three difference values for each of the twelve sensing spots, that is, a 36-dimensional response vector for every sample. The whole of this vector was used in the subsequent statistical analyses.

### 2.4. Statistical Analysis

Principal component analysis (PCA) and hierarchical cluster analysis (HCA) were performed on the response data using Statistical Package for the Social Sciences (Version 20; SPSS Inc.; Chicago, IL, USA) software. The thirty-six response variables are all differences in RGB value and therefore share a common scale; they were entered into both analyses as recorded, without standardization, normalization or other transformation, following the procedure of the previous study [17]. For the PCA, the number of components retained was judged from the cumulative percentage of the total variance that they accounted for. For the HCA, the dissimilarity between two response vectors was expressed as the squared Euclidean distance, and the clusters were formed by Ward’s minimum-variance method. Differences between the concentrations of the target compounds were examined by one-way analysis of variance followed by Duncan’s multiple range test, and differences were taken as significant at *p* < 0.05 [17]. The five concentrations were also examined together, as a single multivariate response, by permutational multivariate analysis of variance, with storage time as the factor tested and storage temperature also entering the model; each compound was scaled to unit variance beforehand so that the five contributed equally, and significance was obtained by permutation.

## 3. Results and Discussion

### 3.1. Changes in the Array Response to Coffee Aroma During Storage

#### 3.1.1. Statistical Analysis of Key Aroma in Coffee Using CSA

Before the stored samples were examined, a separate experiment was carried out to establish whether the array gives distinguishable responses to aroma compounds of different chemical classes. Four key odorants of coffee, furfuryl alcohol, 2,3-butanedione, 2,3-pentanedione and dimethyl trisulfide, were selected for this experiment and were measured individually as pure standards rather than in coffee. They were chosen so that the classes of compound for which the array is formulated are represented alongside a compound belonging to neither: two diketones for the carbonyl-reactive group, one sulfur compound for the group formulated for sulfur volatiles, and furfuryl alcohol, which falls outside both reactive classes. Furfuryl alcohol has been associated with bitter and burnt aromas [32]. 2,3-butanedione and 2,3-pentanedione have been proposed as indicators for aroma freshness of coffee brews. Additionally, 2,3-butanedione and 2,3-pentanedione have been reported to decline in coffee, and this decline was dependent on storage period [11,12,13]. Also, these flavors convey a buttery odor [3,33]. Furthermore, the classes of carbonyl compounds such as 2,3-butanedione and 2,3-pentanedione are distinct from furfuryl alcohol and dimethyl trisulfide.

**Figure 1:**
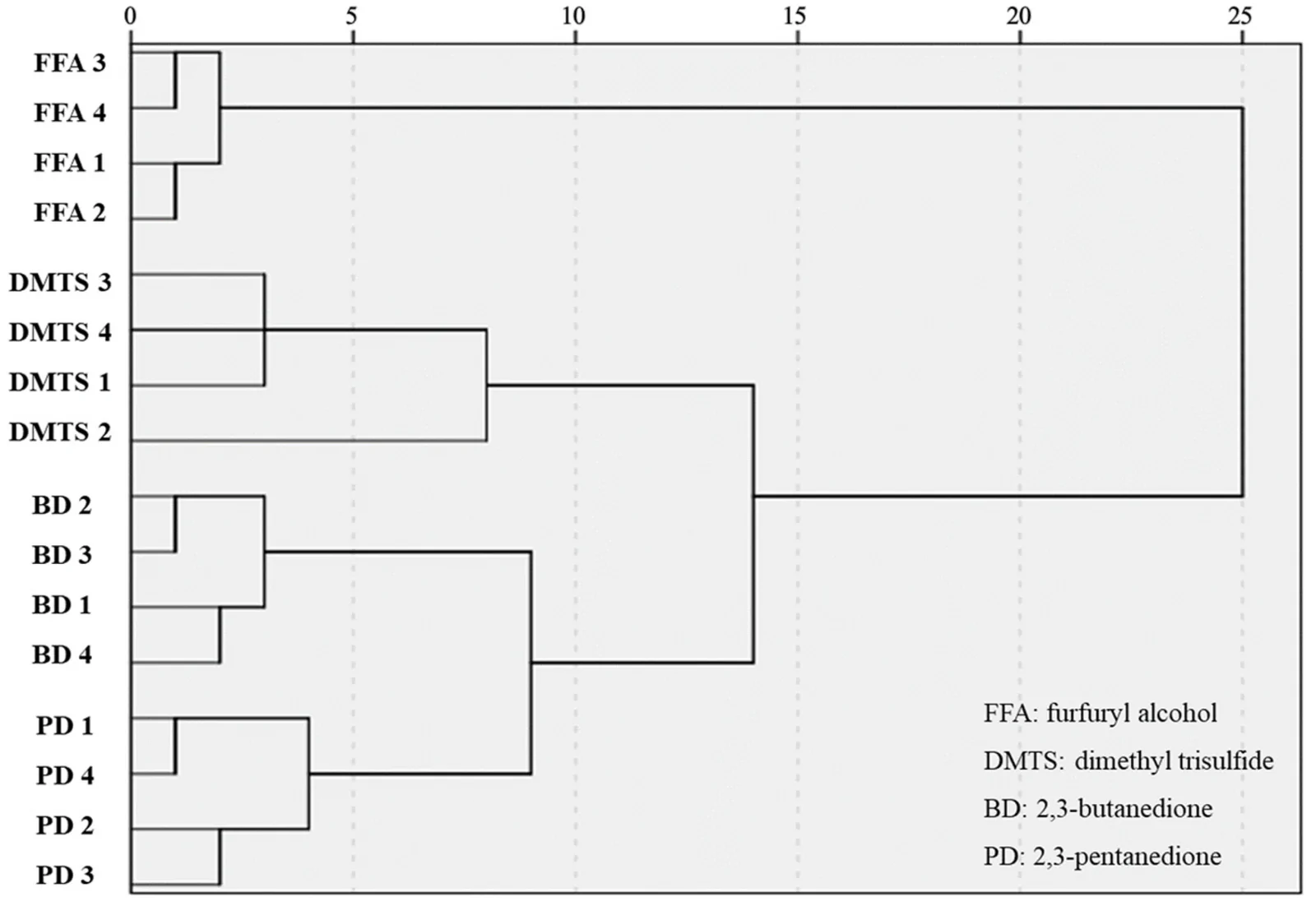
Hierarchical cluster analysis dendrogram of the CSA responses to the four aroma compounds, each measured in four replicates. FFA, furfuryl alcohol; DMTS, dimethyl trisulfide; BD, 2,3-butanedione; PD, 2,3-pentanedione. The four compounds give response patterns that are distinguishable from one another.

HCA groups high-dimensional data and represents the result as a dendrogram. Ward’s minimum-variance method was used. Clusters are formed by minimizing the sum of the squared Euclidean distances (SEDs) from the group centroid to each of the vectors. These clusters are then combined into larger ones, and the procedure is repeated until a single supercluster remains. An HCA dendrogram shows the degree of similarity of the array responses among the various analytes quantitatively using the data’s full dimensionality. It also makes visible any overlap between individual measurements of different analytes. The dendrogram therefore describes how the measurements used to construct it are related to one another; placing a sample that was not used to construct it would require a model validated on independent data. As shown in the HCA dendrogram of the four compounds (Figure 1), each compound was measured in four replicates, and the four replicates of a given compound formed a branch of their own, with no replicate placed in the branch of another compound. The order in which the branches merge also reflects the chemistry of the analytes. The clusters of 2,3-butanedione and 2,3-pentanedione join one another before either joins any other compound, as expected from their common diketone structure and their similar response on the spots formulated for carbonyl compounds. Dimethyl trisulfide joins this pair at a greater distance. Furfuryl alcohol remains separate until the highest level of the dendrogram, so its response pattern is the most distinct of the four. These responses were obtained in the separate experiment described above; the behavior of the array towards the stored coffee samples is considered in the following section.

#### 3.1.2. Statistical Analysis of Coffee Aroma During Storage by CSA

**Figure 2:**
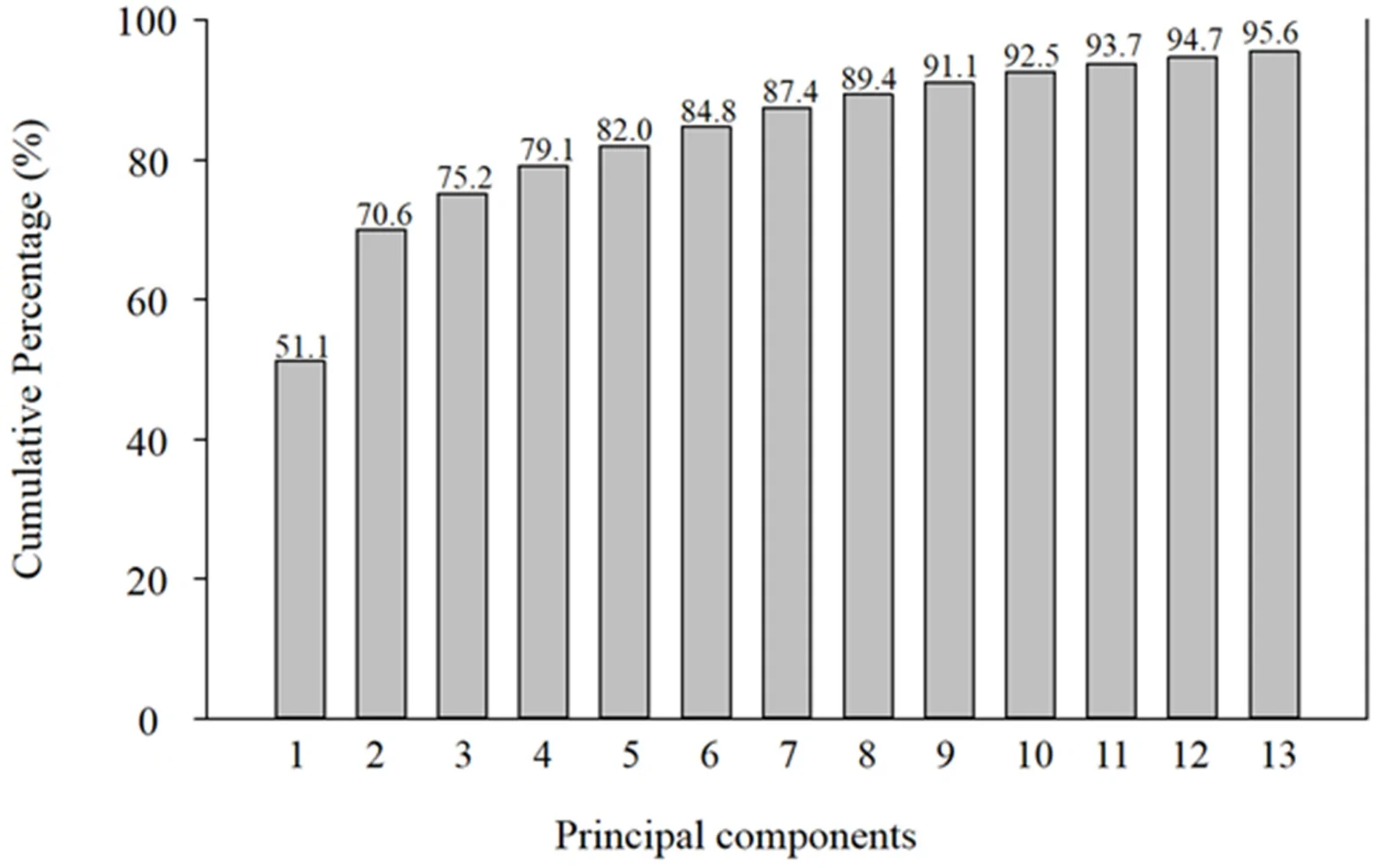
Cumulative percentage of the total variance accounted for by the principal components from PCA.

As shown in Figure 2, 95% of all the data variance is contained in thirteen dimensions. The first principal component alone accounts for 51.1% of the total variance, and the first two together for 70.6%, and the cumulative percentage rises gradually thereafter. Taken by difference, these cumulative values show that each component beyond the first two accounts for a small share of the variance, which is why thirteen components are required to reach 95%. The thirty-six variables are also not independent of one another, since the three color channels recorded at a given sensing spot describe the same color change, so that the effective dimensionality of the response is lower than the nominal figure. Those first two components are the axes of the score plots, which display the separation between the samples themselves. The analysis rests on the sixty array measurements of the storage trial, each of which constitutes a thirty-six-dimensional response vector. The ratio of sixty measurements to thirty-six variables is low, so that the sample covariance matrix, and with it the direction of the latter components, is determined imprecisely; the interpretation of the score plots is therefore confined to the first two components. Because the components are obtained from the measured data by an orthogonal transformation, with no parameter estimated against an outcome, the analysis is not subject to overfitting in the sense of a description that fits these data more closely than data not used to obtain it.

**Figure 3:**
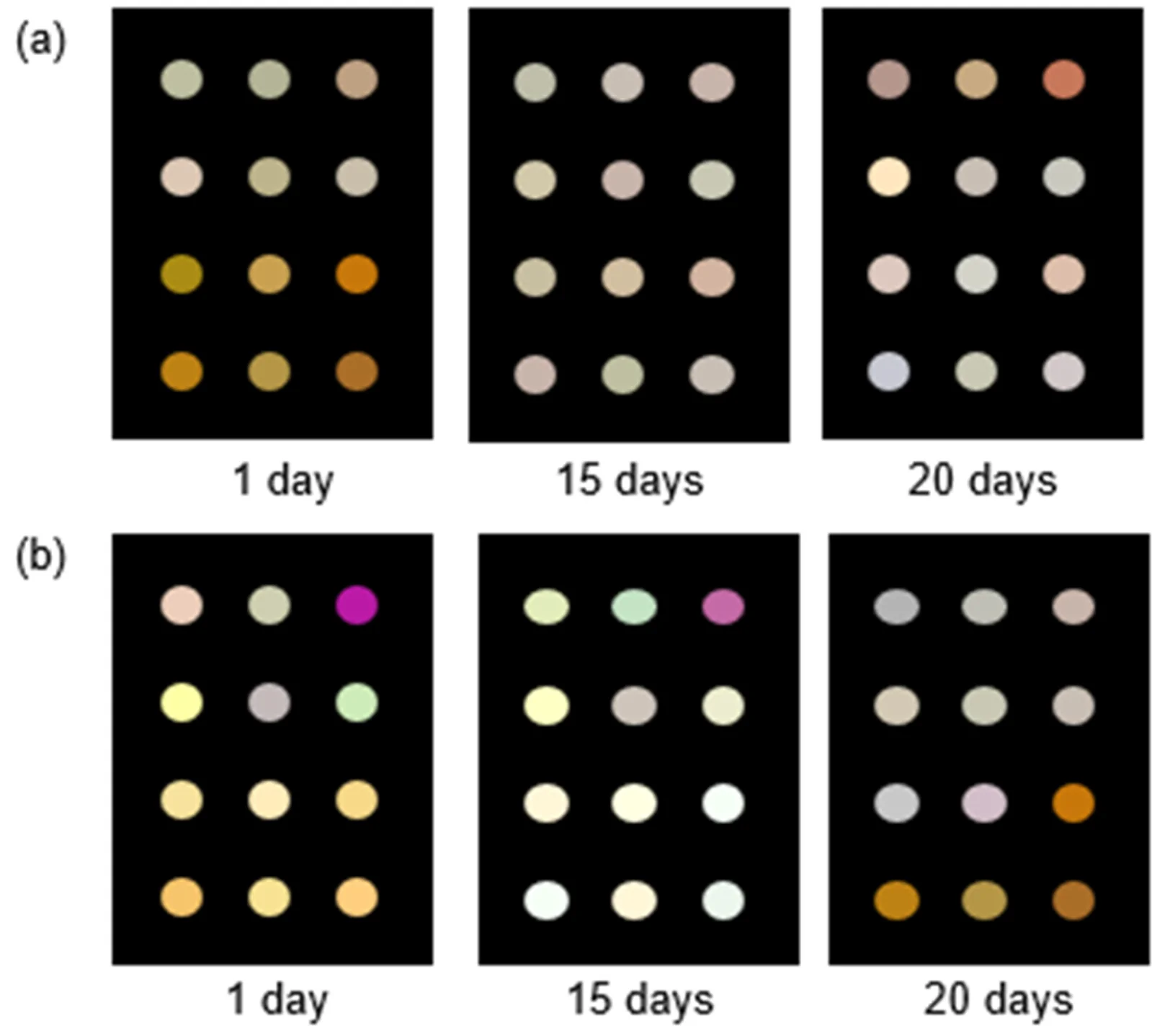
Difference images of CSA for coffee samples during storage at different temperatures: (**a**) 4 °C; (**b**) 25 °C. Each image shows the change in the RGB values of the array on exposure to the sample taken on the day indicated, obtained by subtracting the image recorded before exposure from the image recorded after exposure. The upper six spots are the DNPH-based group and the lower six the DTNB-based group. The days shown were selected so that the progression of the color change is visible to the eye.

**Figure 4:**
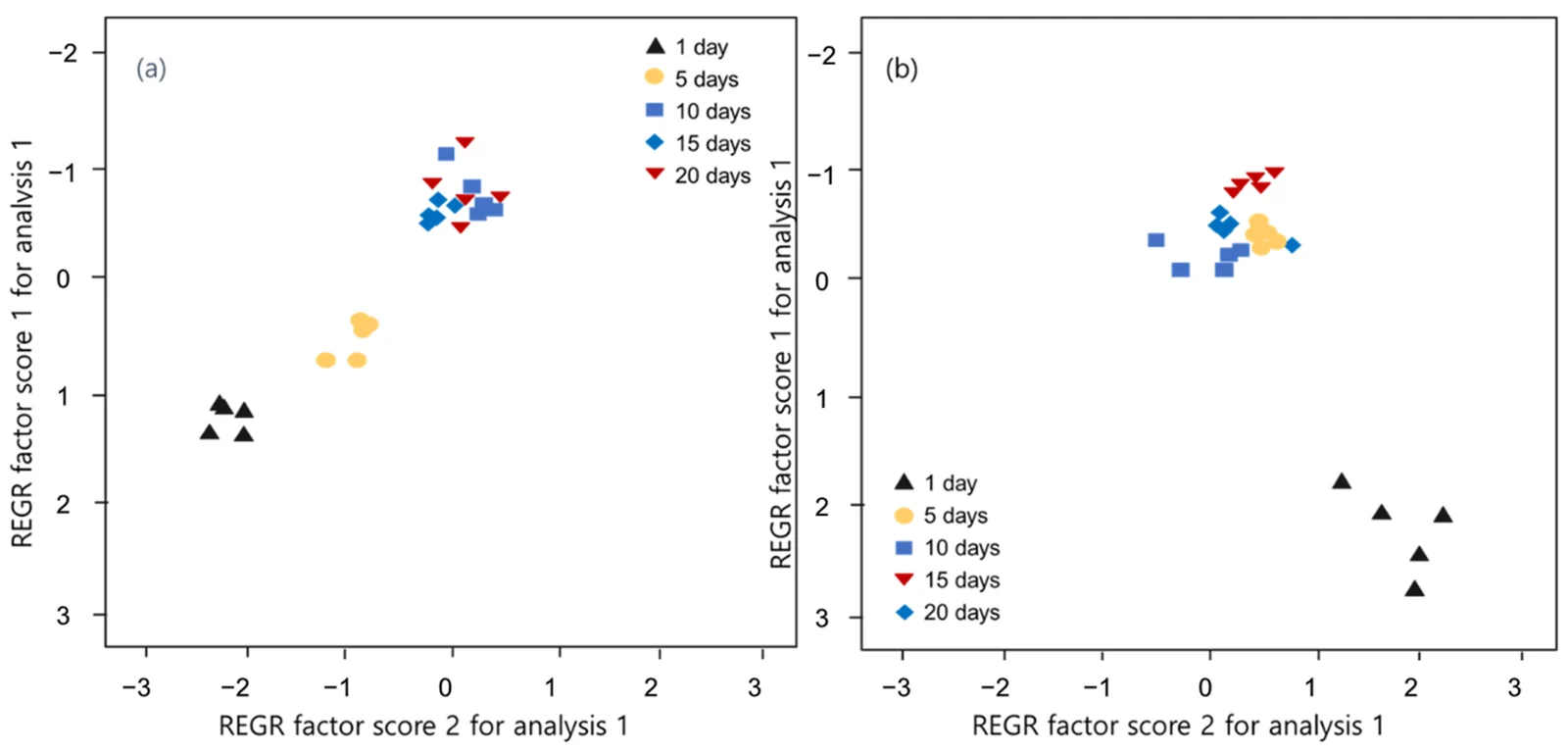
Principal component analysis of coffee samples during storage at different temperatures: (**a**) 4 °C; (**b**) 25 °C. The axes are the scores of the first two principal components, and each point is an individual measurement. The days shown are 1 to 20.

The individual difference images showed patterns that differed with storage temperature and period (Figure 3); how far the samples are separated at each sampling point is addressed by the score plots of Figure 4. This indicates that the array response changes as the aroma of the coffee changes during storage. Each panel of Figure 3 is a difference image: the value shown at each spot is obtained by subtracting the RGB values recorded for that array before exposure from those recorded after exposure, within the same measurement. The colors therefore represent the direction and magnitude of the change at each spot, and not the color of the spot itself, so that a spot which changes little appears dark. The top six sensing spots belong to the first volatile compound group targeted at aldehydes and ketones, while the bottom six spots belong to the second volatile compound group targeting thiols and sulfides. The two groups are formulated for different classes of compounds, DNPH reacting with carbonyl groups and DTNB with free thiols. Every spot, however, also contains a pH indicator, so that its response is not confined to the class its reagent targets: volatiles that alter the local acidity of a spot contribute as well. The response at a given spot therefore reflects several classes of compounds rather than a single molecule, and it is the pattern across the twelve spots, and not the response of any one spot, that distinguishes the samples. The sensing materials of the second group showed faster and more pronounced color changes than those of the first group. The difference images are presented for visual interpretation only; the multivariate analyses were performed on the underlying 36-dimensional response vectors.

The same response data are presented as PCA score plots in Figure 4. At 4 °C (Figure 4a), the samples are arranged in order of storage time, with those from day 1 and day 5 each occupying a region of their own, and those from day 10 onward lying close together. At 25 °C (Figure 4b), the day 1 sample is set well apart from the others, and the samples from day 5 onward lie close together. The two score plots therefore differ in the storage time from which the samples converge, day 10 in Figure 4a and day 5 in Figure 4b. Each point is an individual measurement, five having been obtained at every storage time and temperature, so that the scatter of the replicates can be seen in the figure itself. The day-25 samples were also measured, but their responses were not distinguishable from these later groups and are therefore not shown in Figure 4; this is consistent with the samples becoming less distinguishable from one another as the storage period lengthens.

### 3.2. Changes in the Concentrations of Major Aroma Compounds During Storage by GC-MS

**Figure 5:**
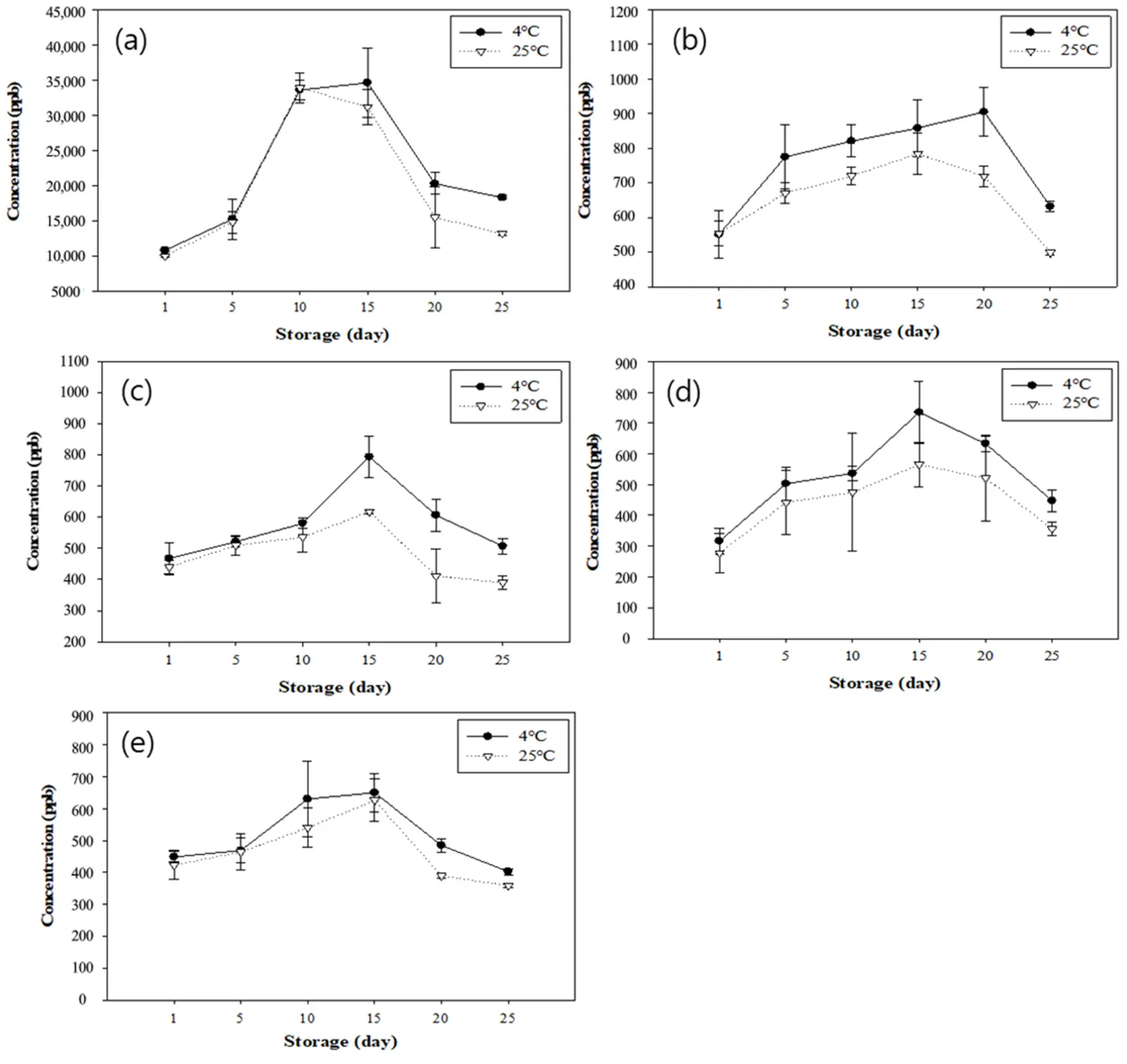
Changes in volatile compounds in coffee samples during the storage period. (**a**) furfuryl alcohol; (**b**) 2-methylbutanal; (**c**) 2,3-butanedione; (**d**) 2,3-pentanedione; (**e**) 2-ethylpyrazine.

Changes in the main aroma compounds of coffee during the storage period were determined by GC-MS. As shown in Figure 5a, the concentration of furfuryl alcohol increased until day 15 and thereafter decreased sharply. The concentration of 2-methylbutanal increased slightly and then decreased after day 20 (Figure 5b); for the samples stored at 4 °C, it did not differ significantly between day 5 and day 20, whereas in the score plot for the same samples (Figure 4a), the day-15 sample occupies a different region from the day-5 sample. In Figure 5c, 2,3-butanedione also increased in concentration and then decreased. There was no significant difference in the concentration between day 1 and day 10. When stored at 25 °C, Figure 5c showed a slight difference in the concentration change between day 1 and day 5, while the samples were separated in the corresponding score plot (Figure 4b). As shown in Figure 5d, 2,3-pentanedione also showed increased concentration and then after 20 days decreased. For storage at 4 °C, no significant increase in concentration was observed between day 5 and day 10, whereas the corresponding samples are shown in Figure 4a. Finally, in Figure 5e, there was no significant difference between samples in 2-ethylpyrazine. The change in the concentration from day 1 to day 5 was not significant when stored at 4 °C. When the five key aroma compounds of coffee were analyzed by GC-MS, they showed a tendency to increase and then to decrease. Taken together as a single multivariate response, the five concentrations differed significantly between storage times (*p* < 0.01), with storage time accounting for about 70% of their total variation.

A similar tendency of variation in aroma concentration has been reported for roasted almonds by Franklin et al. [34], in which alkylpyrazines, Strecker aldehydes, furfural and 2,3-pentanedione increased as a result of lipid oxidation. In their study, 5- to 9-carbon aldehydes and organic acids, 5- to 8-carbon alcohols, 7- and 8-carbon ketones, and 2-pentylfuran were also produced, which caused a paint-like or solvent flavor. In addition, other researchers examined the temporal variations in aroma profiles of coffee samples after grinding, which they attributed to reduced emission of Strecker aldehydes and diketones. The intensity of the earthy and smoky notes also increased over storage [35,36]. These reports are consistent with lipid oxidation contributing to the increases observed here in furfuryl alcohol, 2-methylbutanal, 2,3-butanedione, 2,3-pentanedione and 2-ethylpyrazine. Loss of volatiles through the aroma valve of the package may likewise contribute to the subsequent decline in concentration. Both are proposed as explanations consistent with the observations rather than as mechanisms established here.

The two measurements describe different quantities. The array returns a single multivariate response to the headspace as a whole, whereas the GC-MS analysis gives the concentrations of five individual compounds. A separation between samples in a score plot need not be accompanied by a change in the concentration of an individual compound, since the array response integrates contributions from across the headspace. The two sets of results are accordingly compared as trajectories. Over the period covered by both measurements, the array response shifts furthest over the first half of the storage period and changes little thereafter, whereas the measured concentrations continue to rise to a maximum between day 15 and day 20 and then decline. GC-MS identifies and quantifies the individual compounds, while the array provides a rapid indication of the state of the aroma as a whole.

The rise and fall shown by all five compounds is not described by a monotonic decay. The behavior is better regarded as formation and loss proceeding together, the maximum marking the point at which loss overtakes formation. Fitting a consecutive formation–degradation model would be the way to describe this behavior quantitatively.

Several limitations of the present approach should nevertheless be noted. The CSA does not by itself measure concentration. It reports a pattern of color changes across the twelve spots, from which the overall state of the aroma can be read but not the amount of any individual compound. Relating the array response to the concentration of an individual compound would require the array to be calibrated against concentration, which further work would need to carry out. The trial covered a single dark-roasted Robusta coffee held in aluminum packages with an aroma valve, two storage temperatures and a single 25-day storage period sampled at six time points, so the behavior reported here cannot be assumed to hold for other species, roast degrees or packaging formats. The coffee was roasted in two runs that were combined before grinding, so that the trial provides no replication at the level of the roasting batch. Each package was opened at every sampling point and resealed immediately after the material was withdrawn. These repeated brief exposures to air accompany storage time, and the two cannot be separated; the changes reported are to be read as the combined effect of storage and of the openings that sampling required [13]. The measurements reported here are the array response and the concentrations of five marker compounds. Roast color was not determined, so roast degree is characterized by the roasting conditions used rather than by a color measurement. The ground coffee is likewise characterized by the grinding conditions used. The moisture content and water activity of the roasted beans and of the ground coffee at the start of storage, the headspace oxygen and carbon dioxide, markers of lipid oxidation and sensory assessment lie outside the scope of the measurements made. The observed change is therefore described rather than attributed to a single process, and the array response is not presented as a measure of perceived quality.

## 4. Conclusions

The aroma of a ground coffee of fixed roast does not remain constant during storage. The array response changed with storage time at both storage temperatures, the change being largest over the earlier part of the 25-day period and small thereafter, while the concentrations measured by GC-MS rose to a maximum and then declined rather than decaying monotonically. The aroma of a stored coffee is therefore a state defined by its storage time rather than a fixed property of the roast, and a single rapid array measurement can follow that state alongside the targeted quantification of individual compounds by GC-MS.

The change did not proceed in the same way at the two storage temperatures. The samples ceased to be distinguishable from one another earlier at 25 °C than at 4 °C, from day 5 rather than from day 10, so that the distinctions between successive sampling times persisted longer under refrigerated storage. Storage temperature therefore governs the rate at which the aroma of a stored coffee moves away from its initial state, and a storage time does not define that state without the temperature at which the coffee was held.

Four gaps follow from these results. First, thresholds of acceptability defined by sensory evaluation independently of the instrument are required before an array measurement can be used to estimate shelf life. Second, markers of lipid oxidation measured alongside the aroma compounds would test the mechanism proposed here for the rise in concentration. Third, a consecutive formation–degradation model of the form C(t) = A[exp(−k_2_t) − exp(−k_1_t)] would describe the rise and fall quantitatively, but its rate constants require more sampling beyond the concentration maximum than the present schedule provides, and an apparent activation energy requires more than two storage temperatures. Fourth, two applications remain to be tested: whether a difference in bean grade composition displaces the response pattern from that of the declared product, which requires sets of coffees of known grade composition [37], and whether a position in the response space can be referred to a consumer preference score obtained from a panel stratified by age and gender.

## Data Availability

The original contributions presented in this study are included in the article. Further inquiries can be directed to the corresponding authors.

## Abbreviations

The following abbreviations are used in this manuscript:

CSA	colorimetric sensor array
DNPH	2,4-dinitrophenylhydrazine
DTNB	5,5′-dithiobis(2-nitrobenzoic acid)
GC-MS	gas chromatography–mass spectrometry
HCA	hierarchical cluster analysis
HS-SPME	headspace solid-phase microextraction
PCA	principal component analysis
PEG-400	polyethylene glycol-400
PTFE	polytetrafluoroethylene
PVDF	polyvinylidene fluoride
